# Dating Applications as HIV Prevention Ecosystems in the Era of Digital Sexual Networks

**DOI:** 10.3390/idr18050106

**Published:** 2026-09-17

**Authors:** Francesco De Maria, Paolo Fusco, Alessandro Russo

**Affiliations:** Infectious and Tropical Diseases Unit, Department of Medical and Surgical Sciences, “Magna Graecia” University of Catanzaro, 88100 Catanzaro, Italy; francescodemaria16@gmail.com (F.D.M.); paolofusco89@gmail.com (P.F.)

**Keywords:** HIV prevention, digital medicine, dating applications, artificial intelligence, sexual health, digital health ecosystems, Infectious diseases

## Abstract

Dating applications have become central components of contemporary sexual networks, yet HIV prevention remains largely organised outside these digital environments. We propose that dating applications should be conceptualised as potential HIV and sexual health prevention ecosystems, connecting testing, PrEP and PEP navigation, STI services, vaccination, telemedicine, partner notification, behavioural support, and artificial intelligence-enabled health navigation. Existing evidence supports the effectiveness of many of these digital interventions individually, while emerging integrated models suggest opportunities to connect digital engagement with real-world care pathways. Artificial intelligence may provide an additional navigation layer capable of improving personalisation and continuity across prevention services. However, implementation will require careful attention to privacy, governance, equity, healthcare accessibility, and differences across health systems. Rather than replacing existing healthcare services, digital prevention ecosystems could complement them by bringing prevention closer to the environments where sexual interactions increasingly originate.

## 1. Introduction: The Digitalisation of Sexual Networks

The ways in which people meet sexual partners have changed profoundly over the past two decades. Interactions once mediated primarily through physical social spaces—including bars, clubs, community venues, and personal networks—have increasingly shifted to digital environments. Dating applications and geosocial networking platforms now play a central role in partner seeking, particularly among men who have sex with men (MSM), where they have become deeply embedded within contemporary sexual networks [1,2,3,4].

Although much of the HIV-related literature on dating applications has focused on MSM and sexual minority populations, app-mediated partner seeking is not confined to these groups. Dating applications are also widely used by heterosexual men and women, although patterns of use, motivations, sexual behaviours, and associated health outcomes may differ across populations. A recent systematic review and meta-analysis identified sex-related differences in dating app use and app-mediated sexual and health outcomes, highlighting the diversity of populations engaging in digital sexual networks [5].

This transformation has important implications for HIV prevention. Geosocial networking applications have been associated with increased opportunities for partner acquisition and different patterns of sexual risk behaviour, while users frequently express willingness to engage with sexual health interventions delivered through these platforms [1,2,3,4].

Yet HIV prevention has not fully adapted to the environments in which sexual interactions increasingly originate. Prevention programmes continue to rely largely on clinic-based services, community outreach, public health campaigns, and standalone digital interventions operating outside the platforms where many individuals meet partners and make sexual decisions. This creates an increasing separation between the environments where HIV risk is generated and those where prevention is delivered.

Historically, public health responses have sought to bring prevention to the places where transmission occurs, including bars, clubs, bathhouses, universities, and community settings. As sexual interactions increasingly move online, the question is no longer simply whether digital interventions are effective, but whether prevention is being delivered within the environments where sexual networks are formed.

In this View-Point, we argue that dating applications should be recognised not solely as tools for partner seeking but as potential HIV and sexual health prevention ecosystems. Such environments could connect HIV and STI testing, pre-exposure prophylaxis (PrEP), vaccination, partner notification, behavioural support, and artificial intelligence (AI)-enabled navigation. We therefore propose a shift from isolated digital interventions toward an ecosystem approach that better aligns prevention delivery with contemporary sexual networks.

## 2. The Paradox of Modern HIV Prevention

Over the past decade, digital health has become one of the most rapidly expanding areas of HIV prevention. Mobile applications, social media campaigns, telemedicine services, self-testing programmes, adherence tools, electronic reminders, and AI-based interventions have demonstrated promising results across multiple stages of the prevention continuum [6,7,8,9,10,11,12,13,14,15,16,17,18,19,20].

At first glance, this expansion might suggest that HIV prevention has already entered the digital era. However, digital interventions have often been developed around specific components of the prevention continuum rather than as part of an integrated system. HIV testing, PrEP navigation, adherence support, sexual health education, telehealth, and behavioural interventions have largely been evaluated as individual or targeted strategies, with integration across services remaining an important implementation challenge [21,22].

This reflects the historical development of digital HIV prevention, in which interventions were typically designed to address specific challenges such as testing uptake, adherence, linkage to care, or stigma. Although these approaches can be effective individually, users may still need to navigate separate platforms for self-testing, PrEP information, telemedicine, adherence support, or sexual health education. How these services might function as interconnected components of a broader prevention infrastructure remains less well explored [6,7,8,9,10,11,12,13,14,15,16,17,18,19,20,22].

This fragmentation is particularly relevant when viewed through the lens of digital sexual networks. Dating applications are already environments where users search for partners, communicate about sexual preferences, disclose HIV status or PrEP use, assess risk, and negotiate sexual encounters. Prevention-related decisions may therefore occur within these platforms long before an individual interacts with traditional healthcare services.

The challenge facing public health is thus not merely technological, but conceptual. Prevention largely remains an external service that individuals must actively seek out rather than one connected to the environments where sexual decision-making occurs. As digital platforms increasingly shape contemporary sexual networks, bridging this divide may represent the next step in digital HIV prevention.

## 3. What We Already Know: Digital HIV Prevention Works

The effectiveness of digital HIV prevention interventions is no longer a theoretical question. Over the past decade, an increasingly diverse body of evidence has demonstrated that digital technologies can improve multiple components of the HIV prevention continuum, including testing uptake, linkage to care, PrEP initiation, adherence, retention, and health education [6,17,20,23].

One of the earliest and most successful applications has been the promotion of HIV testing. Social media interventions have shown positive effects on testing uptake, linkage to care, and retention [6], while mobile health interventions, app-based self-assessment tools, and digital self-testing programmes have increased engagement among populations that may face barriers to traditional healthcare access [8,16,18,19]. Importantly, these approaches have also been implemented in settings characterised by structural vulnerabilities, including refugee populations and resource-constrained environments [18], suggesting that digital delivery can extend the reach of prevention beyond conventional healthcare settings.

Substantial progress has similarly been achieved in PrEP. Digital interventions have been used to increase awareness, facilitate navigation through care pathways, reduce stigma, improve adherence, and support retention [6,8,9,10,11,12,13,14,15,24,25]. Systematic reviews further support their potential to enhance PrEP uptake and adherence, particularly among populations with high HIV incidence and substantial digital engagement [9,11,12,25].

Digital interventions can also move beyond information delivery. Real-time adherence support, peer navigation, coaching applications, and personalised messaging can provide ongoing behavioural support [7,10,13,14,15], reflecting the longitudinal nature of HIV prevention. Beyond HIV-specific interventions, digital technologies are increasingly used to support self-care, sexual health services, screening, and communication with healthcare providers [17,20].

Taken together, the evidence indicates that digital HIV prevention can support multiple stages of the prevention continuum. The challenge is increasingly not whether individual digital interventions can work, but how they should be organised within an increasingly digital sexual landscape.

Yet most interventions continue to function as individual or relatively discrete solutions. Comparatively less attention has been devoted to connecting these tools across the prevention continuum. Integrated online-to-offline models are now being developed and evaluated, although evidence supporting comprehensive prevention ecosystems remains limited [22].

## 4. From Digital Interventions to Digital Prevention Ecosystems

The next stage in digital HIV prevention may require moving beyond individual interventions toward a broader ecosystem perspective. In digital health, ecosystem approaches integrate multiple services, stakeholders, and functions within interconnected environments supporting continuity of care [26]. A similar perspective may now be relevant to HIV prevention.

Dating applications already function as social environments in which users identify partners, communicate about sexual preferences and health, assess risk, and make decisions relevant to HIV exposure. Rather than serving solely as targets for prevention campaigns, these platforms could therefore provide entry points to interconnected prevention services, embedded within or directly linked to the environments where sexual decision-making occurs.

A digital HIV and sexual health prevention ecosystem could connect HIV self-testing and facility-based testing, STI screening, PrEP and post-exposure prophylaxis (PEP) navigation, vaccination for HPV, hepatitis A, hepatitis B, and mpox, partner notification, behavioural support, telemedicine, linkage to care, and AI-assisted health navigation.

The objective is not to transform dating applications into healthcare platforms, but to use their position within contemporary sexual networks to facilitate access to prevention. This perspective also extends beyond HIV: dating-app use intersects with the epidemiology and prevention of other STIs, supporting a broader sexual health approach linking HIV prevention with STI screening, vaccination, partner notification, and appropriate care [27].

There is already evidence supporting the plausibility of this approach. Dating-app users have expressed support for sexual health features including testing, partner notification, prevention information, and healthcare navigation [3,4,6], and dating applications have been used to promote HIV self-testing and engagement with prevention services [4,16]. Pilot work has also explored peer-led interventions for safer dating-app use [28], while integrated online-to-offline models linking digital engagement with HIV prevention and treatment services are being formally developed and evaluated [22].

**Figure 1:**
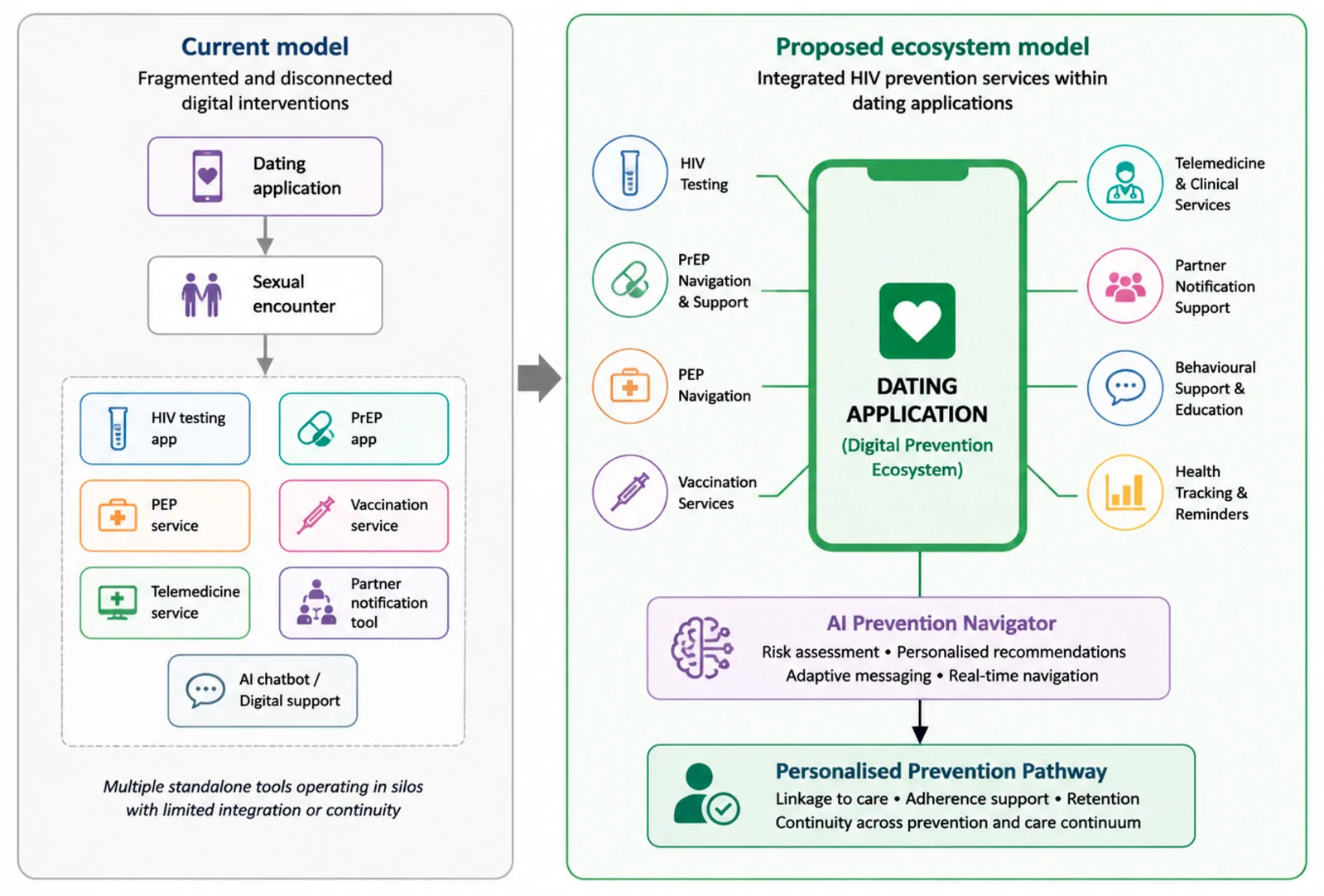
Conceptual transition from fragmented digital HIV prevention interventions to integrated prevention ecosystems embedded within dating applications. Dating applications may evolve from simple partner-seeking platforms into digital prevention ecosystems that integrate testing, PrEP and PEP navigation, vaccination, telemedicine, behavioural support, partner notification, and AI-assisted health navigation within a single digital environment.

The novelty of the ecosystem approach therefore lies not in its individual components, many of which already exist, but in connecting them within or directly to the digital environments where sexual interactions increasingly originate. This transition from individual digital interventions to an integrated prevention ecosystem is illustrated in Figure 1.

## 5. Artificial Intelligence as the Integrative Layer

If dating applications are to evolve into HIV prevention ecosystems, one question remains: what mechanism could connect the various components of such a system into a coherent and personalised user experience? Artificial intelligence may represent one possible answer.

Over the past several years, AI has moved from a theoretical concept to a practical tool in healthcare. Within HIV prevention, machine learning algorithms, predictive analytics, conversational agents, and automated decision-support systems are increasingly being explored for risk assessment, testing promotion, PrEP delivery, adherence support, and healthcare navigation [29,30,31,32,33,34].

Early applications focused primarily on identifying individuals at increased risk of HIV acquisition and improving the targeting of prevention services [29,30,31]. More recently, AI-enabled systems have begun to interact directly with users through conversational interfaces. Chatbots and virtual assistants have shown promise in sexual health education, behavioural counselling, stigma reduction, screening promotion, and engagement with prevention services [34,35,36,37,38,39,40,41,42,43,44].

Within a prevention ecosystem, however, the potential role of AI extends beyond individual interventions. Current prevention pathways may require users to navigate separate services for HIV testing, PrEP eligibility, vaccination, appointments, and adherence support. AI could instead function as a navigation layer connecting these components within digital environments.

For example, an AI-enabled prevention assistant within a dating application could support HIV risk assessment, provide information on testing options, identify potential PrEP eligibility, recommend relevant vaccinations, facilitate appointment scheduling, and deliver personalised reminders. Recommendations could also adapt as individual circumstances change, providing a more responsive experience than static educational materials.

This model is increasingly plausible. Studies have demonstrated the feasibility and acceptability of AI-based interventions in HIV prevention and sexual health [34,35,36,37,38,39,40,41], while reviews suggest an expanding role for AI across prevention, clinical care, health communication, and public health decision-making [29,30,31,32,33,45].

**Figure 2:**
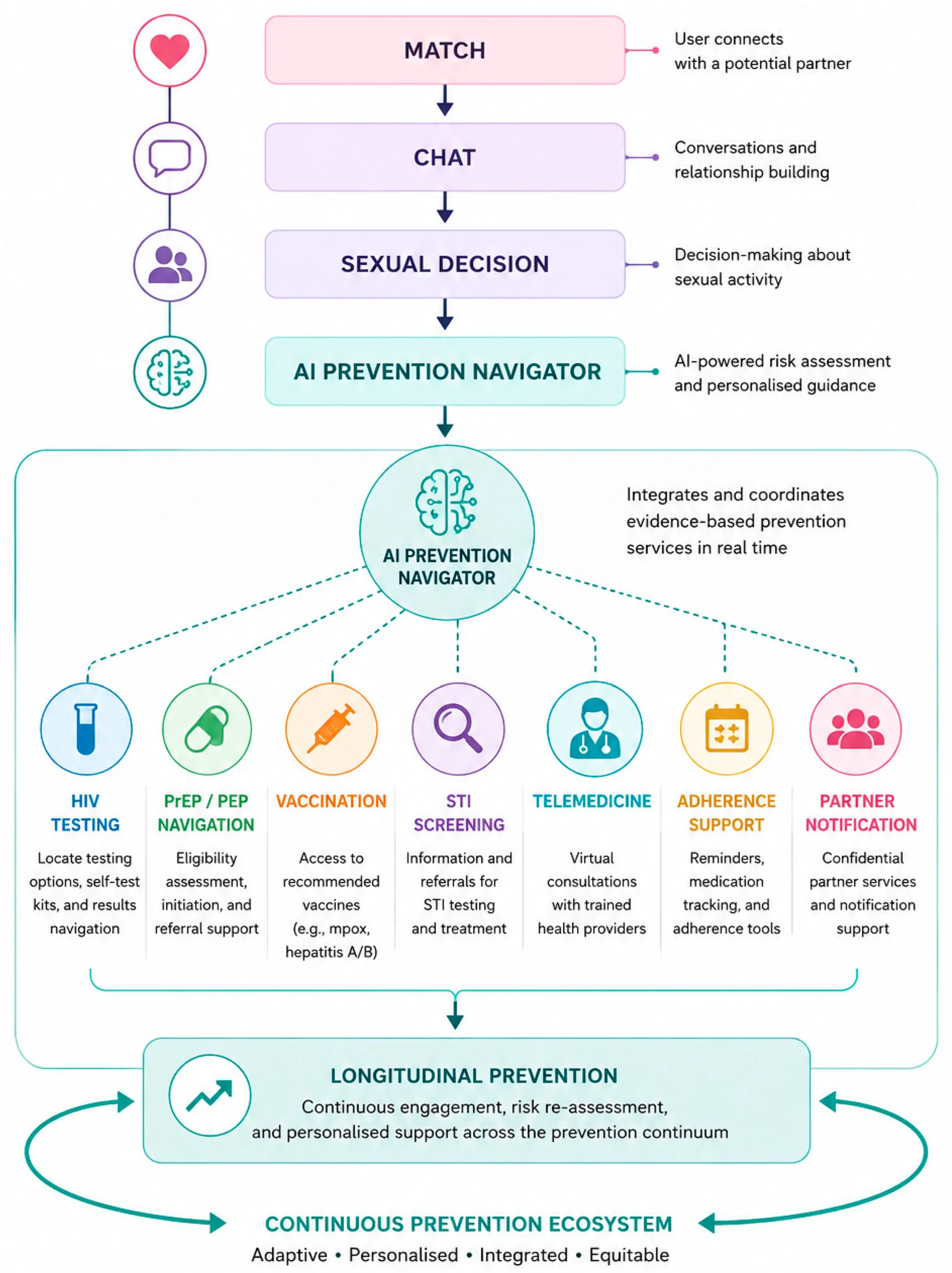
From Match to Prevention: an AI-enabled prevention journey within a dating application ecosystem. The proposed framework illustrates how dating applications could evolve from partner-seeking platforms into integrated prevention ecosystems. Artificial intelligence acts as a navigation layer linking testing, PrEP and PEP services, vaccination, STI screening, telemedicine, adherence support, and partner notification within a continuous and personalised prevention pathway.

AI should therefore be viewed not as a replacement for healthcare providers or as an intervention in itself, but as an enabling layer that could improve navigation, personalisation, and accessibility. By linking testing, PrEP and PEP services, vaccination, STI screening, telemedicine, adherence support, and partner notification, it could help create a more continuous prevention pathway. A conceptual example of this AI-enabled prevention journey is illustrated in Figure 2.

## 6. Ethical, Governance, and Equity Challenges

The integration of HIV prevention services within dating applications raises important ethical, regulatory, and societal questions. The success of any prevention ecosystem will depend not only on technological feasibility but also on the trustworthiness of the environments in which it operates.

Privacy is perhaps the most immediate concern. Dating applications routinely manage sensitive information related to sexual behaviour, identity, location, relationships, and health, and the incorporation of prevention services could further increase the sensitivity of data generated within these platforms. Engagement with health-related services therefore depends on confidence that personal information will not be misused or disclosed without consent [3,33]. Transparent governance, robust data protection, and clear user control over health-related information will be essential.

Platform governance presents a related challenge. Dating applications are typically operated by private companies whose priorities may not always align with those of public health. Sustainable implementation will therefore require collaboration between public health institutions, healthcare providers, technology companies, and affected communities, together with clear accountability for the accuracy of information, recommendations, and management of adverse outcomes.

Equity is equally important. Digital interventions may expand access among populations facing geographical, social, or structural barriers to healthcare, but may also reproduce existing inequalities related to digital literacy, internet access, language, disability, socioeconomic status, or cultural context [18,46]. AI systems introduce additional concerns when the datasets used for their development inadequately represent diverse populations, making inclusion, representativeness, and fairness central considerations [46].

Implementation will also vary considerably across healthcare systems and geographical settings. Telehealth, digital referral pathways, home-based testing, and remote follow-up are well integrated in some settings but remain constrained elsewhere by infrastructure, workforce capacity, connectivity, regulation, and the availability of linked health services. Experience from digital health ecosystems in Africa and telehealth in HIV care illustrates both the opportunities and the context-dependent barriers to implementation [47,48]. The ecosystem proposed here should therefore be understood as an adaptable framework rather than a uniform model.

Digital access alone also does not guarantee access to prevention. Identifying a need for HIV or STI testing, PrEP, PEP, vaccination, or follow-up has limited value if the corresponding service is unavailable or unaffordable. Insurance coverage, public financing, out-of-pocket costs, eligibility requirements, and service availability may determine whether digital engagement translates into effective prevention. Integrated online-to-offline approaches offer one model for connecting digital entry points with existing healthcare pathways [22]. Prevention ecosystems should therefore be designed around linkage to accessible services rather than digital engagement alone.

These challenges extend beyond HIV prevention and are increasingly relevant across digital health as ecosystem-based approaches and AI-enabled services evolve [26,43]. Ethical safeguards should therefore be viewed not as obstacles to innovation but as prerequisites for sustainable implementation. Ultimately, public trust will be as important as technological capability in determining whether prevention ecosystems can achieve meaningful public health impact.

## 7. Conclusions

The digitalisation of sexual networks represents a new challenge for HIV prevention. As partner seeking and sexual decision-making increasingly occur within digital environments, dating applications may provide an opportunity to connect prevention with the settings in which these interactions already take place.

We propose that dating applications should be viewed not solely as platforms for partner seeking, but as potential HIV and sexual health prevention ecosystems. Such ecosystems would not replace healthcare systems or community-based prevention, but could complement them by connecting users with testing, PrEP and PEP, STI services, vaccination, behavioural support, telemedicine, and appropriate care pathways. Although discussed primarily in the context of HIV, this framework may also support a broader approach to sexual health prevention.

Realising this potential will require careful attention to privacy, governance, equity, accessibility, and trust. The future of HIV prevention may depend less on developing new technologies than on integrating existing ones into the places where people already meet, interact, and make decisions about their sexual health.

## Data Availability

No new data were created or analyzed in this study. Data sharing is not applicable to this article.
